# Increasing medicinal and phytochemical compounds of coneflower (*Echinacea purpurea* L.) as affected by NO_3_^−^/NH_4_^+^ ratio and perlite particle size in hydroponics

**DOI:** 10.1038/s41598-021-94589-4

**Published:** 2021-07-26

**Authors:** Fatemeh Ahmadi, Abbas Samadi, Ebrahim Sepehr, Amir Rahimi, Sergey Shabala

**Affiliations:** 1 Department of Soil Science, Faculty of Agriculture, Urmia University, Urmia, Iran; 2 Department of Plant Production and Genetics, Faculty of Agriculture, Urmia University, Urmia, Iran; 3 Tasmanian Institute of Agriculture, University of Tasmania, Hobart, TAS 7001 Australia

**Keywords:** Biochemistry, Environmental sciences

## Abstract

Medicinal plants are considered as one of the most important sources of chemical compounds, so preparing a suitable culture media for medicinal plant growth is a critical factor. The present study is aimed to improve the caffeic acid derivatives and alkylamides percentages of *Echinacea purpurea* root extract in hydroponic culture media with different perlite particle size and NO_3_^−^/NH_4_^+^ ratios. Perlite particle size in the growing media was varied as very coarse perlite (more than 2 mm), coarse perlite (1.5–2 mm), medium perlite (1–1.5 mm), fine perlite (0.5–1 mm), and very fine perlite (less than 0.5 mm) in different ratios to peat moss (including pure perlite, 50:50 v/v, 30:70 v/v, and pure peat moss). Two NO_3_^−^/NH_4_^+^ ratios (90:10 and 70:30) were tested in each growing media. All phytochemical analyses were performed according to standard methods using high performance liquid chromatography (HPLC). It was found that the *E. purpurea* grown in the medium containing very fine-grade perlite with 50:50 v/v perlite to peat moss ratio had the maximum caffeic acid derivatives, including chicoric acid (17 mg g^−1^ DW), caftaric acid (6.3 mg g^−1^ DW), chlorogenic acid (0.93 mg g^−1^ DW), cynarin (0.84 mg g^−1^ DW), and echinacoside (0.73 mg g^−1^ DW), as well as, alkylamides (54.21%). The percentages of these phytochemical compounds increased by decreasing perlite particle size and increasing of NO_3_^−^/NH_4_^+^ ratio. The major alkylamide in the *E. purpurea* root extract was dodeca-2E, 4E, 8Z-10 (E/Z)-tetraenoic acid isobutylamide in all treatments, ranging from 31.12 to 54.21% of total dry weight. It can be concluded that optimizing hydroponic culture media and nutrient solution has significant effects on *E. purpurea* chemical compounds.

## Introduction

The *Echinacea purpurea*, widely known as purple coneflower, is one of the popular medicinal plants of the Asteraceae family, from United States, Canada, Russia, and Australia. In recent years, it has gained international popularity due to claims that it beneficially stimulates the body’s immune system^1,2^. Extracts from the plant have shown antioxidative, antibacterial, antiviral, and antifungal properties, and are used in the treatment of the common cold, as well as respiratory and urinary diseases^3,4^. Caffeic acid derivatives, namely caftaric acid, chlorogenic acid, cynarin, echinacoside, and cichoric acid, are the main compounds of *Echinacea* spp. Of the caffeic acid derivatives, cichoric acid has been shown to possess immunostimulatory properties, promoting phagocyte activity both in vitro and in vivo. Besides, cichoric acid has exhibited antihyaluronidase activity, and a protective effect on the free radical-induced degradation of collagen. Cichoric acid has also been shown to have antiviral activity^5,6^ and, recently, inhibit HIV-1 integrase and replication^7,8^. Echinacoside does not contribute towards the immunostimulant activity, but prospects collagen against reactive oxygen species, and also has antioxidant^9^, anti-inflammatory, and cicatrizing activities^10^. Caftaric acid and chlorogenic acid play an important role in antiviral activity^11^, an effective free radical scavenging agent and preserver of collagen from free radical-induced degradation^10^. Therefore, caffeic acid derivatives are widely measured as markers to determine the medicinal quality of *E. purpurea* extracts^12^. The alkylamides, as a group of bioactive compound, has attached the most interest in terms of pharmacological activity^13^. Plants containing alkylamides are used as spices for their pungent and tingling sensations and are incorporated into topical cosmetics for their wrinkle-smoothing and anti-aging properties^14^. There is increasing evidence that lipophilic *Echinacea* preparations containing *N*-alkylamides can suppress stress-related cellular immune responses^15^. It was also demonstrated recently in several studies that alkylamides -containing *Echinacea* preparations trigger effects on the pro- inflammatory cytokines^16^. Thus, it seems necessary to pay attention to effective strategies to improve the quality of *E. purpurea* phytochemical compounds.

Different cultivation strategies have been developed for the production of *E. purpurea*. However, there are many considerations for plant production in greenhouse conditions, especially in hydroponic (or soilless) culture systems in recent years^17^. Growing in hydroponic media may offer several advantages over field cultivation by controlling the plant nutrition requirement and facilitating the growth conditions^18,19^. Meanwhile, using artificial substrates in the hydroponic cultivation system reduce the cost of establishing advanced cultivation systems, and it enables the farmer to make practical use of it by using commonly raw materials such as cocopeat, sand, and vermiculite as an initial plant growing media^5,20^. Nevertheless, different inorganic and organic products such as peat moss, perlite, mixed materials, etc., are fully or partially used instead of initial substrates due to their useful physical properties^20^. The particle size of substrates is a critical factor in air and water-holding capacity, root distribution, and plant growth, which are different based on their origin and preparation conditions^7,21^. A high volume of roots can concentrate at the top portion of the container includes low aeration and high water-holding capacity^20^.

Attention to the chemical quality of hydroponic nutrient solution and its effect on yield and active compounds is so important^22^. Of these nutrients, two major inorganic forms of nitrogen (N), the ammonium (NH_4_^+^) and the nitrate (NO_3_^−^), can differentially impact plant growth in many plant species^23^. Although the assimilation and metabolism of NH_4_^+^ form require less energy than that of NO_3_^−^ in plants, the majority of plant species grow better on NO_3_^−^ since NH_4_^+^ is toxic for plants and a few species grow well if NH_4_^+^ is the only source of N. It has been reported that using a mixture of NO_3_^−^ and NH_4_^+^ is optimum for nitrogen nutrient in most of the crops^23^. Previous research demonstrated that the NO_3_^−^/NH_4_^+^ ratio could affect the morphological properties of *E. purpurea*^18,24^. Zheng et al.^23^ and Ahmadi et al.^24^ reported that increasing NO_3_^−^ concentration in nutrient solution increased total phenolic and flavonoid contents of *E. purpurea* root extract. However, the plant species and environmental conditions are two critical factors that affect the optimum NO_3_^−^/NH_4_^+^ ratio^25^. So, the present research is focused on the development a hydroponic culture media with various perlite particle sizes and different NO_3_^−^/NH_4_^+^ ratios for improving the caffeic acid derivatives and alkylamides compounds of *E. purpurea* root extract at greenhouse conditions.

## Material and methods

### Growing conditions

**Figure 1:**
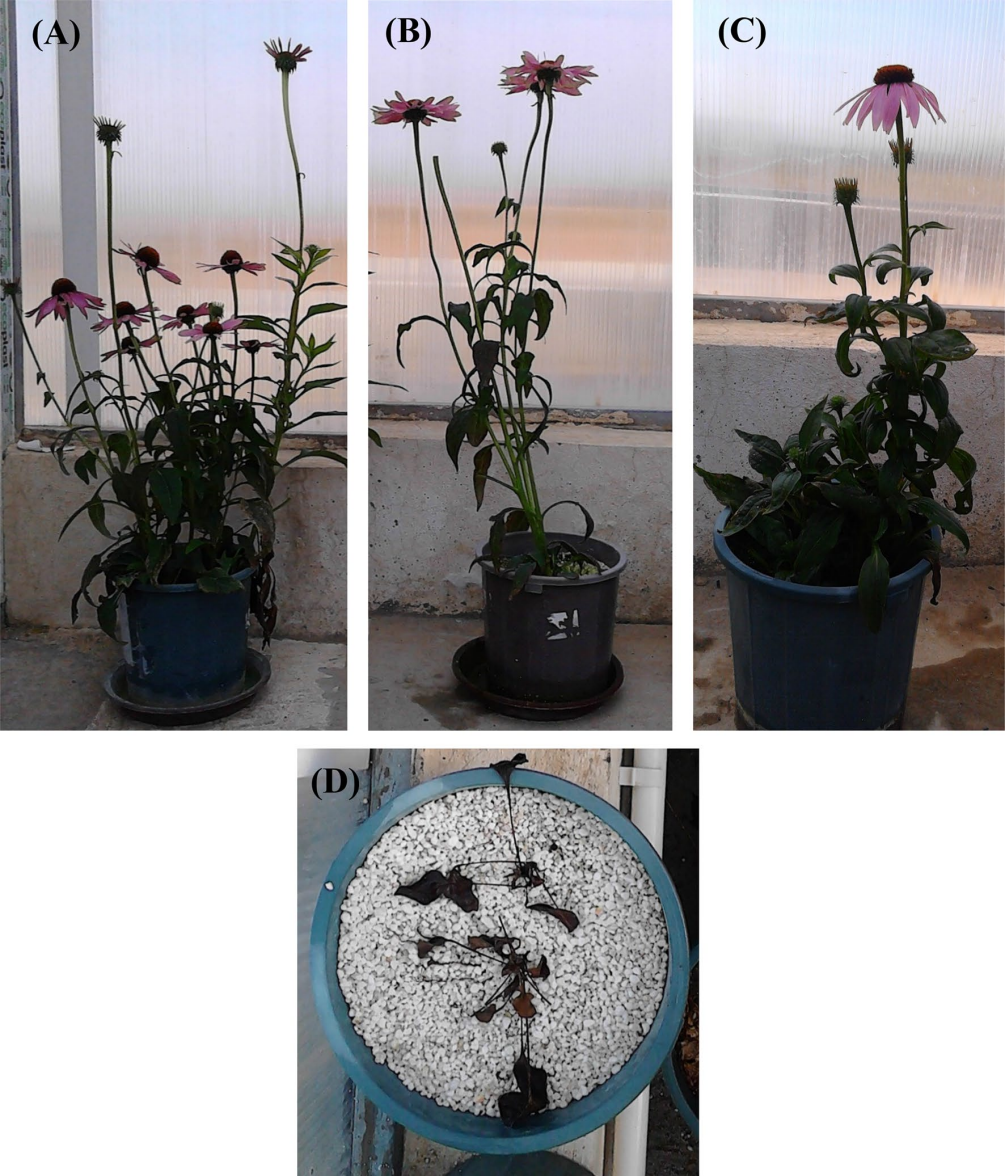
*Echinacea purpurea* grown in (**A**) 50% peat moss + 50% perlite (< 0.5 mm), (**B**) 30% peat moss + 70% perlite (< 0.5 mm), (**C**) 100% peat moss, and (**D**) 100% perlite (> 2 mm) culture media, just at 90:10 NO_3_^−^/NH_4_^+^ ratio (all photos were taken by F. Ahmadi).

The experiment was accomplished in the research glass greenhouse at Urmia University, Iran. The plant growth conditions were controlled regularly inside the glass greenhouse (temperature, humidity, and photosynthetic photon flux density (PPFD) were 22/18 (day/night) ± 1 °C, 70 ± 2%, and 650 ± 2 μmol m^−2^ s^−1^ respectively, and the length of the lighting period was 10 h photoperiod). All greenhouse conditions were controlled during the plant growth. The *E. purpurea* seeds were purchased from an Iranian private joint-stock company, Pakan Bazr Esfahan (www. Pakanbazr.com; plant identification code: OTF-3). Three seeds number were sowed in plastic cups filled with a mixture of perlite and peat mass substrates as a medium to initiate germination. Irrigation was performed based on greenhouse conditions regularly. Seedlings (with four real leaves) were translocated to experimental plastic pots (2.5 L) containing a different ratios of perlite and peat mass as artificial substrates (100% perlite (> 2 mm or > 10 U.S. mesh), 100% peat moss, 50% (v) perlite + 50% (v) peat moss, 70% (v) perlite + 30% (v) peat moss) with various perlite particle size containing less than 0.5 mm (< 35 U.S. mesh), 0.5–1 mm (35–18 U.S. mesh), 1–1.5 mm (18–14 U.S. mesh), 1.5–2 mm (14–10 U.S. mesh), and more than 2 mm (> 10 U.S. mesh). The chemical composition of the nutrient solution based on Zheng et al.^23^ is shown in Table 1. The pH and electrical conductivity (EC) of the nutrient solution were maintained between 5.7–6.2 and 1.0–1.5 dS m^−1^, respectively. According to the plant growth stage, 0.5–3.5 L day^−1^ was used in irrigation system^23^. The *E.purpurea* grown at different culture media at the flowering stage were shown in Fig. 1.

**Table 1:** Chemical properties of nutrient solution (Zheng et al.^23^).

Element	Fertilizer type	Concentration
Nitrogen (N)	(NH_4_)_2_SO_4_-KNO_3_-Ca(NO_3_)_2_	15 mM
Phosphorus (P)	H_3_PO_3_	1 mM
Potassium (K)	KNO_3_	6 mM
Calcium (Ca)	Ca(NO_3_)_2_	4 mM
Magnesium (Mg)	MgSO_4_‧7H_2_O	2 mM
Sulfur (S)	Sulfate fertilizers	2 mM
Iron (Fe)	Fe-EDTA	50 µM
Manganese (Mn)	Mn SO_4_‧H_2_O	9 µM
Copper (Cu)	CuSO_4_‧5H_2_O	0.3 µM
Zinc (Zn)	ZnSO_4_‧7H_2_O	0.8 µM
Boron (B)	H_3_BO_3_	15 µM
Molybdenum (Mo)	H_24_Mo_7_N_6_O_24_‧4H_2_O	0.11 µM

### Sample preparation

Plants were harvested at the end of the flowering stage (8 months). The plants were divided into roots, stems, flower heads, and lower and upper leaves after washing with tap water. Root, samples were dried at 25 ± 1^ °^C, ground into a fine powder, and collected for phytochemical analysis^26^. Due to the perennial nature of this plant and the flowering of the plant in the second year, economically, flowers are first considered as a medicinal organ and then the root. Of course, the root is the most important part and organ of medicine that can be grown in bioreactors.

### Total phenolic content

Measuring the total phenolic compounds in root samples was performed by the Folin–Ciocalteau method adapted from Singleton et al.^27^. In details, 10 μL of methanolic extracts and 1600 μL of distilled water was mixed, then 200 μL of Folin–Ciocalteau reagent (10% V/V prepared in distilled water) were added and left at 25 °C for 5 min, then 200 μL of sodium carbonate (7.5%) was added and left in a dark place at 25 °C for 30 min. The absorbance was measured at 760 nm using a spectrophotometer (UV–Visible Spectrophotometer, USA). To quantitative analysis of total phenolic content, the gallic acid was used as an external standard, and total phenolic content was expressed as mg gallic acid g^−1^ DW^27^.

### Caffeic acid derivatives

**Figure 2:**
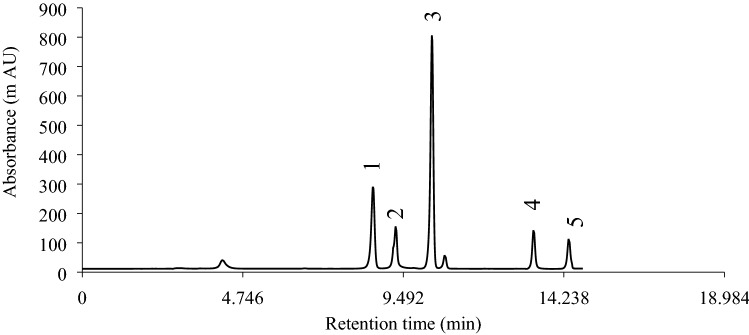
HPLC profile of a standard mixture of caffeic acid derivatives. Peak 1, caftaric acid; 2, chlorogenic acid; 3, chicoric acid; 4 cynarin, and 5, echinacoside.

The phenolic acids analysis was carried out on an Agilent Technologies 1100 series HPLC (Agilent Technologies, Wilmington, DE, USA), equipped with a 20 μL manual sample loop, degasser, quaternary pump, column oven, and diode array detector. The Separation of analytes was performed on the ZORBAX Eclipse XDB column (4.6 mm × 250 mm, 5 μm pore size, Germany), which was thermostatically controlled at 28 °C according to a method adapted from Mei et al.^36^. The extracts were filtered (through a 0.45 μm filter), and then 20 μL was injected into the HPLC–DAD. A gradient elution program was used to separation of phenolic acids, by changing the acetonitrile to the acetic acid proportion (1.0% V/V in water). The initial composition of the mobile phase was acetonitrile and acetic acid (1.0% V/V in water) with a 10:90 portion. Then, the composition of the mobile phase was changed from 10 to 25% acetonitrile for 5 min, from 25 to 65% acetonitrile in 10 min, and remained in this condition for 5 min. After that, the mobile phase composition back to the initial condition for 5 min and kept for another 5 min, before the injection of another sample. The total time of analysis per sample was 30 min. The flow rate was adjusted at 1.5 mL min^−1^, and the wavelengths of detection of phenolic acids and recording of chromatograms were set at 272, 250, 310, and 360 nm. Identification of the phenolic compounds was based on comparing the retention time and diode array spectra of commercially standard compounds with components of real samples. Stock standard solutions of phenolic acids (1000 μg mL^−1^) were prepared by dissolving 10 mg of each analyte in 10 mL liquid chromatographic grade methanol followed by sonication for 10 min, placed in dark-brown vials, and stored in a refrigerator at 4 °C. The content of each phenolic acid in the sample was calculated using the calibration curves equation and the integrated peak area. The HPLC profile of a standard mixture of caffeic acid derivatives is shown in Fig. 2.

### Alkylamides

A sub-sample (1 g) was mixed with 100% methanol for the alkylamides and the mixture sonicated for ten minutes, filtered through Whatman #1 paper, and the liquid extract made up to 100 mL. Considerable effort was devoted to maximizing the amount of constituents extracted^14^. Aliquots (20 μL) were analyzed by HPLC as described. The mobile phase for alkylamide separation was modified from Bauer and Remiger^28^ and utilized an acetonitrile/water gradient commencing at 40% acetonitrile for 10 min followed by a gradient ramp at 1 mL/min to 53% acetonitrile at 35 min). The mobile phase for caffeoyl phenol separation was modified from Bauer (4) and utilized an acidified (1% of 0.1 M H_3_PO_4_) methanol/water gradient commencing at 10% methanol gradient ramp at 1 mL min^−1^ to 50% methanol at 20 min. Quantification was based on the peak area of working reference compounds. The alkylamide working reference compound was trans, trans-2, 4-dodecadienal (Lancaster Synthesis, Eastgate, England), which was initially calibrated against an isomeric mixture dodeca-2,4,8,10-tetraenoic acid isobutyl amide. The alkylamides were quantified using the same response factor for all peaks.

### Statistical analysis

The statistics were based on the factorial with a completely randomized design with three replications. The factors contained different sizes of perlite, including very coarse perlite (more than 2 mm), coarse perlite (1.5–2 mm), medium perlite (1–1.5 mm), fine perlite (0.5–1 mm), and very fine perlite (less than 0.5 mm), two NO_3_^−^/NH_4_^+^ rations (90:10 and 70:30), and a mixture of peat moss with different size of perlite at 50:50 v/v and 30:70 v/v peat moss to perlite ratios and pure perlite and peat moss (100% by volume). Data were analyzed using Duncan's multiple range tests at *P* ≤ 0.01, using SAS (Version 9.4; SAS Institute, 2011) statistical program.

### License for the collection of plant specimen

The authors declare that the collection of plant and seed specimens were according to authorized rules.

### Complying with relevant institutional, national, and international guidelines and legislation

The authors declare that all relevant institutional, national, and international guidelines and legislation were respected.

## Results and discussion

### Phenolic compounds and caffeic acid derivatives

**Figure 3:**
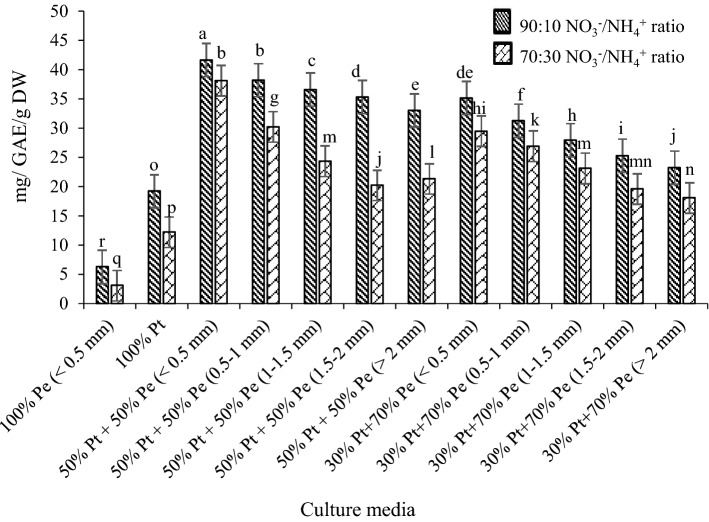
Total phenolic of *E.purpurea* root at different culture media and NO_3_^−^/NH_4_^+^ ratio.

**Figure 4:**
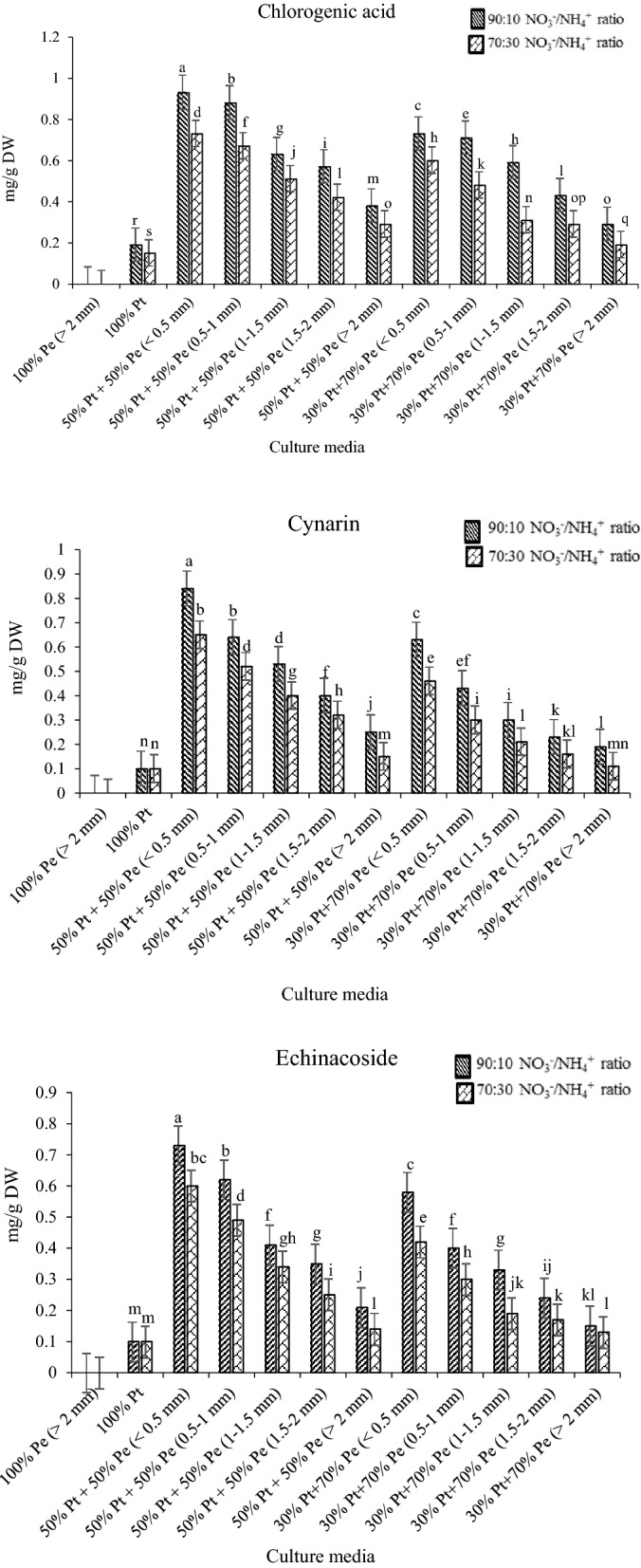

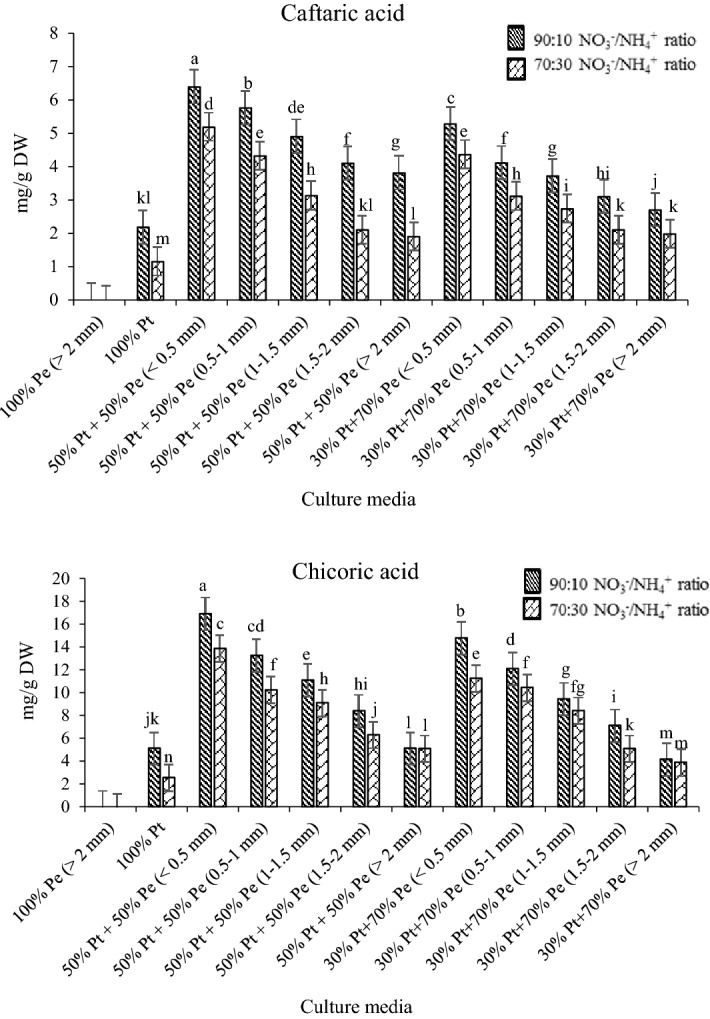
Caffeic acid derivatives of *E.purpurea* root at different culture media and NO_3_^−^/NH_4_^+^ ratios.

Medicinal herbs are often cultivated in soilless cultures, and the quality of phytochemical compounds is strongly affected by the nutrient solution^29^. Results of total phenolics content and caffeic acid derivatives in root samples of *E. purpurea* subjected to different NO_3_^−^/NH_4_^+^ ratios are shown in Figs. 3 and 4, respectively.

Both total phenolics and caffeic acid derivatives were affected by growing media and NO_3_^−^/NH_4_^+^ ratios. The highest root total phenolics (42 mg GAE g^−1^ DW) and concentrations of different caffeic acid deviations in all samples were found in cultivated *E. purpurea* at the 50% perlite + 50% peat mass medium with perlite particle size less than 0.5 mm and 90:10 NO_3_^−^/NH_4_^+^ ratio (Figs. 3, 4). Total phenolic compounds (Fig. 3) and caffeic acid derivatives (Fig. 4) decreased with a decreasing NO_3_^−^/NH_4_^+^ ratio. Meanwhile, increasing the particle size of perlite in culture media decreased the total phenolic compounds (Fig. 3) and caffeic acid derivatives concentration (Fig. 4) in the samples. In general, the dried *E. purpurea* root contained more chicoric acid (max 17 mg g^−1^ DW) and caftaric acid (max 6.3 mg g^−1^ DW) than other compounds in all treatments (Fig. 4). Statistical analysis showed the significant interaction of NO_3_^−^/NH_4_^+^ ratio and perlite particle size on caffeic acid derivatives content. Many studies have shown that the formation and collection of phenolic compounds in various plant organs is a mechanism to cope with the stresses caused by overexploitation of plant nutrients^30^. Based on the morphological characteristics of the *E. purpurea* in different NO_3_^−^/NH_4_^+^ ratio, including leaf shrinkage and severe burn of the leaf margin, especially at 90:10 NO_3_^−^/NH_4_^+^ ratio, which shows the NO_3_^−^ toxicity, it is inferred that plants in higher NO_3_^−^/NH_4_^+^ ratio are affected by NO_3_^−^ stress. It is demonstrated that the biosynthesis of root phenolic compounds caused reduce in nitrate toxicity^31^. There are previous reports^32^ showing that the phenylalanine ammonia-lyase (PAL) activity was increased under stress conditions. The activity of PAL depends on the stage of plant growth^22^. Meanwhile, plant growth conditions, time of harvest, and geographical location affect the PLA activity^17^. The same results were reported by Vidović et al.^30^. The various secondary metabolites are responsible for medically active compounds under plant stress conditions^23^, which the synthesis, accumulation, sequestration, and degradation pathways have not been elucidated^33^.

Plants may produce large amounts of phenolic compounds and phenolic derivatives in the roots in response to exceed nitrate accumulation^34^. According to Ghabaei et al.^12^, one of the mitigating mechanisms of nitrogen excess stress is the accumulation of phenolic compounds in the plant. Therefore, it can be said that creating stress conditions during plant growth conditions is one of the effective ways to increase the active compounds in the plants^18^. Chicoric acid, chlorogenic acid, caftaric acid, cynarin, and echinacoside are the most potential phenolic compounds in *E.purpurea* root extract^23^. Different culture media resulted in different concentrations of these caffeic acid derivatives (Fig. 4). Overall, plants grown on the 50% perlite + 50% peat mass medium with perlite particle size less than 0.5 mm and 90:10 NO_3_^−^/NH_4_^+^ ratio had the highest concentrations of chicoric acid (17 mg g^−1^ DW), caftaric acid (6.3 mg g^−1^ DW), chlorogenic acid (0.93 mg g^−1^ DW), cynarin (0.84 mg g^−1^ DW), and echinacoside (0.73 mg g^−1^ DW). It found that the concentration of chicoric acid was more than other derivatives in all culture media, which significantly decreased by increasing perlite particle size and decreasing NO_3_^−^/NH_4_^+^ ratios. The same trend was found for other derivatives (Fig. 4). The difference in the phenolic acid concentrations extracted from the plant roots depends on various factors such as environmental growth conditions (light, temperature, and humidity) and especially the growing period of the plant^34^. It is well known that the concentrations of caffeic acid derivatives is different during vegetative to reproductive stages^35^. According to Karg et al.^35^, a significant increase in chicoric acid concentration was obtained at the end of the reproductive stage. However, the chlorogenic acid, echinacoside, and cynarin content were significantly reduced in the period^33^. Accordingly, the determination of caffeic acid deviations at the end of the flowering stage at the present study found more chicoric acid and caftaric acid concentrations than others. According to Lema-Rumińska et al.^2^, the chicoric acid and caftaric acid concentrations in the plant root extract were 5.3 and 1.5 mg g^−1^ DW, respectively. In other research^36^, the concentrations of chicoric acid and caftaric acid in the root of *E. purpurea* extract were 8.3 and 2.2 mg g^−1^ DW, respectively. However, the chicoric acid and caftaric acid concentrations in the present study were found maximum 17 and 6.9 mg g^−1^ DW respectively at the 50% perlite + 50% peat mass medium with perlite particle size less than 0.5 mm and 90:10 NO_3_^−^/NH_4_^+^ ratio, which indicates the influence of growing media and NO_3_^−^/NH_4_^+^ ratio on the plant phytochemical parameters.

The antiviral activity and protective effects of chicoric acid on the free radical-induced collagen degradation were reported^25^. The improving of immunity and antioxidant effects of *E.purpurea* is correlated with polyphenols compound such as caftaric acid. Among all caffeic acid derivatives, caftaric acid and chlorogenic acid can act an extreme antioxidants against free radicals^37^. Echinacoside and caffeic acid have antiviral and antibacterial effects against reactive oxygen species^38^. According to the results, the maximum echinacoside and cynarin concentrations of *E. purpurea* root extract were significantly more than previous reports^39,40^. Increasing echinacoside concentration, as an effective phytochemical compound and secondary metabolite of *E. purpurea* is so important in pharmacognosy and herbal researches^34^. It was found that echinacoside could prevent the progress of neurodegeneration in Parkinson’s and Alzheimer’s diseases^38^. Based on the effect of cultivation method on the caffeic acid derivatives content, which are essential substrates for production and biosynthesis of different immunological drugs, the previous researches showed that the total root content of caftaric acid, chlorogenic acid, and chicoric acid in open-field cultivation method were 4.35, 5.02, and 28.06 mg g^−1^ DW, respectively^41^. However, in vitro cultivation of *E. purpurea* in basal medium (MS-medium) with 3% sucrose and 0.7% agar showed the maximum 3.66 mg g^−1^ DW caffeic acid content^42^. Comparing of the obtained results in this study with previous researches indicates the efficiency of hydroponic cultivation system for increasing of caffeic acid derivatives of *E. purpurea*.

### Alkylamides

High performance liquid chromatography investigation of the alkylamide constituents revealed the presence of 11 alkylamides in the *E. purpurea* root extract (Table 2); however, the percentage of the alkylamides are widely different in the plants grown on different culture media and NO_3_^−^/NH_4_^+^ ratios (Tables 3, 4).

**Table 2:** Alkylamide composition of *E. purpurea*.

Compound	Rt^a^ (min)	Constituents
1	13.38	Undeca, 2E,4Z-diene-8,10-diynoic acid isobutylamide
2	15.73	Undeca, 2Z,4E-diene-8,10-diynoic acid isobutylamide
3	16.25	Dodeca-2E, 4Z-diene-8,10-diynoic acid isobutylamide
4	17.92	Dodeca-2E, 4Z-diene-8,10-diynoic acid 2-methylbutylamide
5	19.35	Dodeca-4E, 10E-trien-8,10-diynoic acid 2-methylbutylamide
6	20.09	Trideca, 2E, 7Z-diene-10, 12-diynoic acid isobutylamide
7	20.80	Dodeca-2E, 4Z-diene-8,10-diynoic acid 2-methylbutylamide
8	23.90	Dodeca-2E, 4E, 8Z-10 (E/Z)-tetraenoic acid isobutylamide
9	24.10	Dodeca-2E, 4E, 8Z,10 (E/Z)-tetraenoic acid isobutylamide
10	28.61	Dodeca-2E, 4E, 8Z-tetraenoic acid isobutylamide
11	30.35	Dodeca-2E, 4E-dienoic acid isobutylamide

^a^Retention time in HPLC system.

**Table 3:** Alkylamides percentage of *E. purpurea* grown at different culture media and 90:10 NO_3_^−^/NH_4_^+^ ratio.

Culture media	Alkylamides (% of total dry weight**)**										
	1	2	3	4	5	6	7	8	9	10	11
100% Pe (> 2 mm)	nd*	nd	nd	nd	nd	nd	nd	nd	nd	nd	nd
100% Pt	2.25	1.42	1.22	Trace	0.81	13.68	Trace	45.91	30.1	1.10	0.66
50% Pt + 50% Pe (< 0.5 mm)	0.81	Trace	0.25	Trace	0.10	Trace	2.11	54.21	39.19	2.33	1
50% Pt + 50% Pe (0.5–1 mm)	4.32	0.74	Trace	0.15	2.35	3	1.65	50.09	35.21	1.58	0.91
50% Pt + 50% Pe (1–1.5 mm)	5.96	0.71	2.19	3.69	Trace	2.17	1.29	48.69	33.12	1.35	0.83
50% Pt + 50% Pe (1.5–2 mm)	9.09	0.68	1.51	2.67	1.09	1.91	1.19	47	32.95	1.11	0.80
50% Pt + 50% Pe (> 2 mm)	15.96	0.66	0.84	Trace	Trace	1.63	0.92	46.19	32.11	0.96	0.73
30% Pt + 70% Pe (< 0.5 mm)	2	1.32	trace	2.19	Trace	1.16	1.90	52.16	36.38	1.96	0.93
30% Pt + 70% Pe (0.5–1 mm)	9.7	1.15	0.81	2.11	0.94	Trace	0.87	49.03	33.29	1.40	0.70
30% Pt + 70% Pe (1–1.5 mm)	16.63	0.92	0.77	Trace	Trace	0.98	0.83	46.20	31.69	1.32	0.66
30% Pt + 70% Pe (1.5–2 mm)	18.06	Trace	0.60	1.13	0.89	0.71	0.72	45.65	30.59	1.05	0.60
30% Pt + 70% Pe (> 2 mm)	22.79	0.63	Trace	Trace	0.83	0.66	0.59	43.19	30.10	0.67	0.54

**Table 4:** Alkylamides percentage of *E. purpurea* grown at different culture media and 70:30 NO_3_^−^/NH_4_^+^ ratio.

Culture media	Alkylamides (% of total dry weight)										
	1	2	3	4	5	6	7	8	9	10	11
100% Pe (> 2 mm)	nd	nd	nd	nd	nd	nd	nd	nd	nd	nd	nd
100% Pt	18.33	1.20	0.98	Trace	0.63	12.30	trace	39.39	25.69	0.91	0.57
50% Pt + 50% Pe (< 0.5 mm)	21.03	Trace	0.27	Trace	Trace	Trace	1.32	43.58	31.69	1.38	0.73
50% Pt + 50% Pe (0.5–1 mm)	23.99	0.62	Trace	0.13	1.62	2.46	1.10	40.36	27.61	1.49	0.62
50% Pt + 50% Pe (1–1.5 mm)	31.75	0.73	1.35	2.51	trace	1.98	0.94	34.69	24.39	1.09	0.57
50% Pt + 50% Pe (1.5–2 mm)	37.18	0.54	1.09	1.16	0.84	1.14	0.84	32.10	23.84	0.87	0.43
50% Pt + 50% Pe (> 2 mm)	39.39	0.48	0.76	Trace	Trace	0.94	0.62	31.69	25.10	0.62	0.40
30% Pt + 70% Pe (< 0.5 mm)	24.09	1.18	Trace	1.59	Trace	0.91	0.91	42.18	27.39	1.14	0.61
30% Pt + 70% Pe (0.5–1 mm)	31	1.01	0.71	1.13	0.72	trace	0.74	37.39	25.97	0.91	0.42
30% Pt + 70% Pe (1–1.5 mm)	39.25	0.81	0.69	Trace	Trace	0.72	0.63	35.40	21.39	0.72	0.39
30% Pt + 70% Pe (1.5–2 mm)	43.37	Trace	0.60	0.94	0.62	0.68	0.53	31.96	20.39	0.66	0.25
30% Pt + 70% Pe (> 2 mm)	45.93	0.59	Trace	Trace	0.48	0.53	0.49	31.12	20.09	0.58	0.19

*nd* not detected (very low percentage).

The highest percentage of different alkylamides were found in the *E. purpurea* grown at 50:50 v/v perlite to peat moss ratio culture media. The *E. purpurea* grown at the culture media with higher perlite volume percentage, had the lowest alkylamides percentages, as the lowest percentage (not detectable) of alkylamides was found in the plants grown in 100% perlite culture medium. Perlite particle size also affected the alkylamides percentages. Increasing the perlite particle size in culture media, leads to a decrease in the *E. purpurea* root alkylamides percentages. The culture media with perlite particle size less than 0.5 mm had more alkylamides percentages than others. More alkylamides percentage were found in the *E. purpurea* root extract by increasing of NO_3_^−^/NH_4_^+^ ratio (Tables 3, 4).

The major alkylamide in the *E. purpurea* root extract was dodeca-2E, 4E, 8Z-10 (E/Z)-tetraenoic acid isobutylamide (isomeric compounds labeled with 8 and 9 in Table 3) in all treatments, ranging from 31.12 to 54.21% of total dry weight, which was varied at different culture media and NO_3_^−^/NH_4_^+^ ratios. The alkylamide percentage was the highest (54.21%) of all at the 50:50 v/v perlite to peat moss ratio with perlite particle size less than 0.5 mm and 90:10 NO_3_^−^/NH_4_^+^ ratio. Decreasing of perlite volume percentage and particle size caused to increase of the alkylamide percentage in *E. purpurea* root extract (Tables 3, 4). In contrast, it was found that the undeca, 2E, 4Z-diene-8, 10-diynoic acid isobutylamide (compound label with 1 in Table 3) had different trend against of other alkylamides, which increased by increasing of perlite volume percentage, particle size, and NO_3_^−^/NH_4_^+^ ratio (Tables 3, 4).

Several isomeric pairs of alkylamides found in *Echinacea*, the structures differing solely by the double-bond configuration^16^. Stuart and Wills (2000) showed that the *E. purpurea* root extract contained 70% of the total plant alkylamides with 20% in flower, and 10% in the stem. They also reported that the dodeca-2E, 4E, 8Z-10 (E/Z)-tetraenoic acid isobutylamide was the main alkylamide in the root extract (32.73% of total dry weight). More than 20 different alkylamides were identified based on their mass spectra in the *E. purpurea* root extract by the LC–MS method^43^. They reported that the total percentage of the dodeca-2E, 4E, 8Z-10 (E/Z)-tetraenoic acid isobutylamide was 36.81%. Based on the fact that the phytochemical profiles of different *Echinacea* products are highly variable and strictly depend on the plant growth stage, Gulledge et al.^16^ reported that the higher percentage of root alkylamides, especially dodeca-2E, 4E, 8Z-10 (E/Z)-tetraenoic acid isobutylamide (30.69%) was found at the flowering stage. Comparing of results of the present study with different previous reports showed the higher percentage of root alkylamides in *E. purpurea* growing at the 50:50 v/v perlite to peat moss ratio with perlite particle size less than 0.5 mm at 90:10 NO_3_^−^/NH_4_^+^ ratio. The percentage of dodeca-2E, 4E, 8Z-10 (E/Z)-tetraenoic acid isobutylamide in this study was more than that reported by other researchers (Tables 3, 4). Dodeca-2E, 4E, 8Z-10 (E/Z)-tetraenoic acid isobutylamide has been shown to induce anti-inflammatory responses in mouse macrophages^3^ interact with the endocannabinoid system^14^ and inhibit COX-2 activity^15^ confirming the in vitro effect of this compound on the immune response.

## Conclusion

In summary, the results demonstrate that hydroponic cultivation of *E. purpurea* at different volume percentages of perlite and peat moss with application of different NO_3_^−^/NH_4_^+^ ratios could affect the phytochemical properties of the plant. Decreasing perlite size and increase of NO_3_^−^/NH_4_^+^ ratio caused a significant increase in total phenolic content and different caffeic acid derivatives concentrations, including chicoric acid, caftaric acid, chlorogenic acid, cynarin, and echinacoside, as well as different alkylamides percentage in *E. purpurea* root extract. The highest concentrations of caffeic acid derivatives and alkylamides percentage found in the medium containing very fine-grade perlite (< 0.5 mm) with 50:50 v/v perlite to peat moss ratio. The percentages of these phytochemical compounds were increased by increasing of NO_3_^−^/NH_4_^+^ ratio. Dodeca-2E, 4E, 8Z-10 (E/Z)-tetraenoic acid isobutylamide was the most predominant alkylamide in the plant root extract. Using perlite and peat moss mixture for plant cultivation not only affects the plant phytochemical compounds, but also reduces production costs in hydroponic systems.
